# Association between Physiological Equivalent Temperature (PET) with adverse pregnancy outcomes in Ahvaz, southwest of Iran

**DOI:** 10.1186/s12884-021-03876-5

**Published:** 2021-06-04

**Authors:** Maryam Dastoorpoor, Narges Khanjani, Narges Khodadadi

**Affiliations:** 1 Department of Epidemiology and Biostatistics, Menopause Andropause Research Center, Ahvaz Jundishapur University of Medical Sciences, Ahvaz, Iran; 2 Environmental Health Engineering Research Center, Kerman University of Medical Sciences, Kerman, Iran

**Keywords:** Temperature, Thermal index, Physiological Equivalent Temperature, Pregnancy Outcome

## Abstract

**Background:**

There are few epidemiological studies on the relation between temperature changes and adverse pregnancy outcomes. The purpose of this study was to determine the relation between Physiological Equivalent Temperature (PET) with adverse pregnancy outcomes including stillbirth, low birth weight (LBW), preterm labor (PTL), spontaneous abortion (SA), preeclampsia and hypertension in Ahvaz, Iran.

**Methods:**

Distributed Lag Non-linear Models (DLNM) combined with quasi-Poisson regression were used to investigate the effect of PET on adverse pregnancy outcomes. In this study the effect of time trend, air pollutants (NO_2_, SO_2_ and PM_10_), and weekdays were adjusted.

**Results:**

High PET (45.4 C°, lag = 0) caused a significant increase in risk of stillbirth. Also, high levels of PET (45.4, 43.6, 42.5 C°, lag = 0–6) and low levels of PET (9.9, 16.9 C°, lags = 0, 0–13, 0–21) significantly increased the risk of LBW. But, low levels of PET (6.4, 9.9, 16.9 C°, lags = 0–6, 0–13) reduced the risk of gestational hypertension.

**Conclusion:**

The results of this study showed that hot and cold thermal stress may be associated with increased risk of stillbirth, and LBW in Ahvaz.

## Background

Weather conditions can affect human health and activities [1]. Heat stress is a major cause of climate-related deaths. As temperatures continue to increase due to climate change, heat stress is expected to worsen [2]; and negative outcomes might increase as a result of human exposure to extreme weather conditions. Researchers think one of the most important weather-related causes of mortality in the developed and developing world, is extreme heat [3]. Previous studies conducted in Iran have reported that the risk of mortality due to extreme heat was higher than other unfavorable temperatures [4]. Studies have reported that heat-related deaths were more prone to occur on days with a peak temperature above 38 °C, and there was an exponential relation between the incidence of these deaths and the number of hot days per year [5].

Recently, several studies have focused on the possible effects of ambient temperature on pregnancy outcomes. For example, Yu et al. (2018) reported mixed effects of temperature on preterm birth, in the tropical island of Puerto Rico. But, other studies have reported a positive relation between exposure to heat waves in all months of pregnancy and preterm birth, while exposure during early pregnancy was more likely to increase the risk of stillbirth, compared with exposure in the last months of pregnancy [6]. Several studies have reported associations between stillbirth and higher temperatures during the week before delivery [7–10]. Other studies have shown the effect of high temperatures on preterm birth [11], gestational diabetes mellitus [12] and birth weight [13]. A 2017 analysis in the US estimated that exposures to extreme ambient temperature including cold exposures during the second and third trimesters, and hot exposures during the third trimester of pregnancy, compared to average temperatures may increase the risk of low birth weight [8]. Another study showed that singleton primiparous women who conceived in summer and had a longer exposure to higher ambient temperature were at a greater risk of preeclampsia [14]. Likewise, other researchers found that rise in temperature 30 days after conception and in the first trimester of pregnancy, increased the risk of severe pre-eclampsia especially when conception had happened in summer [15]. And others claimed that cumulative and acute exposures to extremely low or high temperatures, both may induce emotional stress during pregnancy [16], and increase the risk of hypertension in pregnancy during cold months [17–19].

Human thermal discomfort can be measured by various indices [1]. Since 1950, human thermal comfort in indoor and outdoor environments has been discussed in numerous reports, leading to various numerical and diagram-based indices [20]. Currently, there are more than 60 heat stress indices, which have their own advantages and disadvantages [21]. This indicates that the assessment of heat stress in terms of physiological and psychological strain in humans is complex. The many indices that have been suggested can be categorized into one of these three groups: “rational indices”, “empirical indices”, or “direct indices”. The first and second groups are sophisticated indices, which integrate environmental and physiological variables. They are difficult to calculate and are not feasible for daily use. The last group consists of simple indicators based on the measurement of underlying environmental variables [22].

One of the most popular indices used to measure heat stress in outdoor spaces is physiological equivalent temperature (PET), deviated from the Munich Energy Balance for Individuals (MEMI) [23]. PET is defined as a complete budget model for the heat of the human body (Höppe1984, 1999), and provides the equivalent temperature of an isothermal reference environment with a water vapor pressure of 12 hPa (50 % at 20 °C) and light wind (0.1 m/s), at which the heat balance of a reference person is maintained with core and skin temperature equal to those under the conditions being assessed. For the reference person, an optimal indoor environment is chosen and a work metabolism of 80 W will be added to the basic metabolism and clothing insulation should be fixed at 0.9 Clo. The effect of humidity on PET is limited to the latent heat fluxes via respiration and through diffusion through the skin [24]. The assessment of PET has its roots in Fanger’s (1970) PMV, calculated for different air temperatures in the reference environment, using the settings for the PET reference person [25].

According to numerous studies, the dangers of climate change on human health are undeniable. Although pregnant women are a sensitive group, the extent of these changes during pregnancy is still unknown. The present study is the first study about the effects of temperature on adverse pregnancy outcomes in Iran and the Middle East.

The aim of the present paper is to study the relation between PET and stillbirth, low birth weight (LBW), preterm labor (PTL), spontaneous abortion (SA), preeclampsia and gestational hypertension in a 10 year (2008–2018) time frame in Ahvaz, Iran.

## Methods

### Study site

Ahvaz is the seventh most populous city of Iran. Ahvaz is the capital city of Khuzestan province in the south-west of Iran. Ahvaz is located at 31°20’N and 48°40’E. It’s area is185 km^2^ and is 12 m above sea level. In the 2016 census, the population of this city was approximately 1,300,000 [26]. Ahvaz has a desert climate with hot long summers and short mild winters. Ahvaz is often the hottest city in the world during summer, with high consistent temperatures between 45 and 50 °C. The annual average temperature in this city is 25.4 °C. Ahvaz recorded the temperature of 54 °C which was the highest temperature recorded in the world on June 29, 2017 [27].

### Data

Data about adverse pregnancy outcomes including stillbirth, LBW, PTL, SA, preeclampsia and hypertension (diagnosed in the clinic and recorded with date in the patients files) were collected from the population of pregnant women that visited two big referral hospitals in Ahvaz, named the Imam Khomeini, and Razi Hospital, from 2008 to 2018 (10 years). The diagnoses were based on ICD-10 and included the following codes; stillbirth (Z37.1), low birth weight (P07.0), preterm labor (O60), spontaneous abortion (O03), pre-eclampsia (O14), and gestational hypertension (O13).

The data was inquired on a daily basis, from the beginning of April 2008 until March 2018. The total number of pregnant women who visited the Obstetrics and Gynecology department during this 10-year period was 150,766.

Meteorological parameters including average daily temperature, maximum daily temperature, minimum daily temperature, average wind speed, relative humidity, and cloudiness were obtained from the Khuzestan Meteorological Department. Ahvaz city has one synoptic meteorology station, in which different atmospheric factors such as temperature, type of clouds, rainfall and cloudiness is determined according to specific instructions, and recorded at certain hours, daily. This station is 22.5 m above sea level, at 48°40’E longitude and 31°20’N latitude, inside Ahvaz.

Data about ambient air pollutants were inquired from the Ahvaz Environmental Protection Organization and included SO_2_, PM_10_, NO_2_. There are four air pollution monitoring stations in Ahvaz city and in this study the average of the four stations were used. Missing air pollutants data were estimated using the EM (Expectation-Maximization) method [28].

The Expectation Maximum method uses the available data to create regression models that estimate missing data. In this method, regression parameters are re-estimated several times and updated regularly using new sets. That is, initially the available data is used to estimate the parameters of the model. Then the available data and the estimated data, are used to re-estimate the missing data. This process is repeated until the difference between two consecutive regression coefficients becomes less than 10^− 6^ [28]. In this study, there was no missing meteorological data, but there was less than 10 % missing in air pollution data, that was estimated using EM.

## PET index

The data required to calculate the PET index are 1- Meteorological parameters including air temperature in degrees Celsius (C^°^), relative humidity in percentage (%), wind speed in meters per second (m/s), cloudiness in Oktas (octants), vapour pressure of water in hectopascal (hPa), radiation temperature in degrees Celsius (C^°^), global radiation (G) in Watts per square meter (W/m^2^), 2- Physiological data including height, weight, age, gender, amount of clothing in clo (clo), amount of physical activity in Watts (W), 3- Geographic data including latitude, longitude and altitude of the meteorological measurement stations. Physiological parameters are different in different people. In order to calculate the PET index, the physiological parameters for all people are considered a standard amount; gender is assumed female, height is set at 175 centimeter, weight at 75 kg, age at 35 years, clothing = 0.9 Clo and physical activity level equal to 80 W [29].

Analysis of PET index was performed using the Rayman software. The RayMan software is a simulation tool used in human-biometeorology. Details about this software can be found elsewhere [30].

The classification of PET index in Iran is presented in Table 1 (16, 29). The thermal comfort point for PET is from 17.8 to 27 ° C, at this point there is no thermal stress imposed on humans.

**Table 1:** Thermal sensation and different groups of PET

PET (°C) in Iran^a^	Thermal sensation	Physiological stress level
< -10.7	Very cold	Extreme cold stress
-10.7 to -0.7	Cold	Strong cold stress
-0.7 to 8.8	Cool	Moderate cold stress
8.8 to 17.8	Slightly cool	Slight cold stress
17.8 to 27.0	Comfortable	No thermal stress
27.0 to 35.1	Slightly warm	Slight heat stress
35.1 to 43.0	Warm	Moderate heat stress
43.0 to 50.8	Hot	Strong heat stress
> 50.8	Very hot	Extreme heat stress

^a^Table taken from Sharafkhani et al. [31]

In order to investigate the effect of PET index on study outcomes, two separate analysis were performed. First, the association between low values (comparison of 1, 5 and 25th percentiles relative to no thermal stress) and high values (comparison of 90, 95 and 99th percentiles relative to no thermal stress) of PET index with the risk of adverse pregnancy outcomes, in zero cumulative models, 0–2, 0–6, 0–13 and 0–21 days lag was determined. The median of PET, defined as the no thermal stress class, was respectively 22.5 °C and was used as the basis for comparison with other high and low thermal stress values. Zero cumulative models used in this study was models estimating the effect of exposure from day 0 until a particular day. For example, the 0–2 model, means the effect observed from day 0 (same day, lag = 0) until day 2 (lag = 2 days).

Second, the association between cold thermal stress (comparison of 1th percentile relative to 25th percentile) and hot thermal stress (comparison of 75th percentile relative to 99th percentile) of PET index with risk of adverse pregnancy outcomes, in zero cumulative models, 0–2, 0–6, 0–13 and 0–21 days lag was calculated according to methods used in previous references [31–33].

### Statistical Analysis

In order to investigate the effect of PET on adverse pregnancy outcomes, Distributed Lag Non-linear Models (DLNM) combined with quasi-Poisson regression models were used. The DLNM model, is based on cross-basis functions, and is used for simultaneous estimation of the nonlinear relation between exposure and outcome in different time lags [34].

In this study, a natural cubic-spline DLNM was used to determine the nonlinear relation of PET index and also the cumulative lag effects up to a maximum of 21 days, similar to previous studies [35, 36], with adverse pregnancy outcomes.

Spline knots were set at equally spaced values on the log scale of lags. The long term, seasonal trend of adverse pregnancy outcomes was adjusted by a natural cubic spline function of time with 7 degrees of freedom per years of study (10 years). PM_10_, SO_2_ and NO_2_ were controlled using the stratified distributed lag model for up to 7 days lag with 3 degrees of freedom [31]. Also, the holidays and weekdays variable was adjusted as a categorical variable in the final model [31]. Akaike Information Criterion (AIC) was used to select the most appropriate model and degrees of freedom (knots) for thermal index and lags [34]. Five degrees of freedom were considered as the best model for thermal index and time lags. The risk ratio and 95 % confidence interval were estimated for the associations. The analysis was performed utilizing R software version 3.5.3 through the dlnm package. P-values less than 0.05 were considered significant.

## Results

The descriptive statistics of the PET index, stillbirth, LBW, PTL, SA, preeclampsia and gestational hypertension are presented in Table 2. During the 10-years study period, the highest and lowest adverse pregnancy outcomes were respectively preterm labors (5776 cases) and stillbirths (1965 cases). The mean ± SD of PET was 27.6 ± 11.6 (Table 2).

**Table 2:** Descriptive statistics of adverse pregnancy outcomes, air pollutants, PET in Ahvaz city, 2008–2018

Variable (Mean per day)	N	Mean ± SD	Median	Min	Max
Stillbirth	1965	0.5 ± 0.8	0	0	5
LBW^a^	863	0.2 ± 0.6	0	0	5
PTL^b^	5776	1.6 ± 1.9	1	0	12
SA^c^	5063	1.4 ± 1.4	1	0	8
Pre-Eclampsia	4357	1.2 ± 1.2	1	0	8
Gestational hypertension	4030	1.1 ± 1.2	1	0	13
NO_2_^d^(µg/m^3^)	---	46.4 ± 43.1	35.6	1.5	443.8
SO_2_^e^ (µg/m^3^)	---	48.8 ± 57.0	35.9	0	907.4
PM_10_^f^ (µg/m^3^)	---	216.9 ± 278.3	149.2	1.8	4324.2
PET ^g^	---	27.6 ± 11.6	27.9	0.8	47.3

^a^Low birth weight

^b^Preterm labor

^c^Spontaneous abortion

^d^Nitrogen dioxide

^e^Sulfur dioxide

^f^Particulate matter less than 10 microns

^g^Physiological equivalent temperature

### PET index and adverse pregnancy outcomes

The association between the PET index and adverse pregnancy outcomes in 1st (6.4 ° C), 5th (9.9 ° C), 25th (16.9 °C), 90th (42.5° C), 95th (43.6 ° C) and 99th (45.4° C) percentiles relative to PET = 22.5 °C is presented in Table 3.

**Fig. 1:**
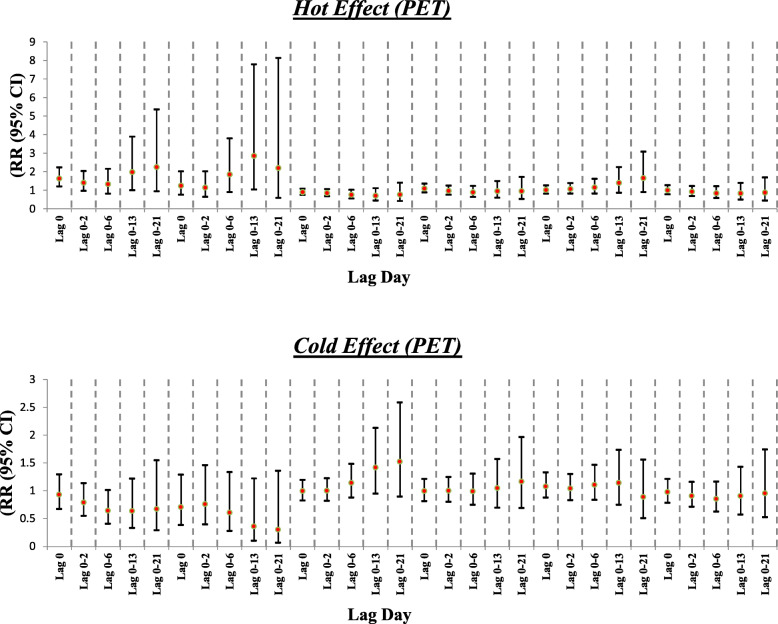
The relative risks (95 % CIs) of hot and cold thermal stress of PET on adverse pregnancy outcomes at different lag days. *Statistically significant.

The results in Table 3 show that high PET values in the 99th percentile compared to no thermal stress, at lag 0 increased the risk of stillbirth significantly. Also in relation to the LBW outcome, both high values of PET in 90th, 95th and 99th percentiles, in the cumulative lag of 0–6 and low values of PET in the 5th and 25th percentiles in 0, 0–13 and 0-21 day lags, compared to no thermal stress, significantly increased the risk of LBW (Table 3). Regarding gestational hypertension, the results showed that low levels of PET in the 1st, 5th, and 25th percentiles compared to no thermal stress, at lags 0–6 and 0–13 reduced the risk of gestational hypertension (Table 3). Significant changes were not observed in the risk of other pregnancy outcomes including PTL, SA and pre-eclampsia, with PET values in any lag (Table 3). The results of intensified thermal stress analysis showed that hot thermal stress (comparing the 99th percentile to 75th percentile) significantly increased the risk of stillbirth in lags 0 and 0–13 and LBW in lag 0–13 (Table 4; Fig. 1).

**Table 3:** The cumulative relative risks of adverse pregnancy outcomes in high and low PET ^a^ values relative to PET = 22.5 °C

Repercussions	PET value(°C)		Lag 0	Lag 0–2	Lag 0–6	Lag 0–13	Lag 0–21
Stillbirth	H^b^	45.4	**1.980 (1.097–3.574)**	1.602 (0.793–3.234)	1.282 (0.505–3.253)	1.747 (0.457–6.676)	1.750 (0.315–9.707)
		43.6	1.667 (0.960–2.895)	1.425 (0.746–2.722)	1.166 (0.501–2.711)	1.396 (0.422–4.616)	1.350 (0.299–6.093)
		42.5	1.515 (0.887–2.587)	1.334 (0.716–2.485)	1.105 (0.496–2.461)	1.228 (0.401–3.763)	1.161 0.288–4.69)
	L^c^	16.9	0.961 (0.810–1.140)	0.956 (0.782–1.168)	0.903 (0.694–1.174)	0.930 (0.638–1.359)	1.116 (0.691–1.802)
		9.9	1.023 (0.734–1.426)	0.888 (0.601–1.311)	0.655 (0.392–1.093)	0.620 (0.298–1.288)	0.770 (0.304–1.955)
		6.4	0.894 (0.606–1.319)	0.752 (0.486–1.163)	0.579 (0.334–1.004)	0.589 (0.270–1.288)	0.747 (0.277–2.016)
LBW	H	45.4	0.918 (0.349–2.416)	1.280 (0.422–3.885)	**4.966 (1.161–21.241)**	7.181 (0.860-59.949)	2.938 (0.192–45.004)
		43.6	0.848 (0.341–2.111)	1.239 (0.443–3.467)	**4.180 (1.099–15.896)**	5.278 (0.764–36.458)	2.367 (0.203–27.641)
		42.5	0.812 (0.334–1.973)	1.213 (0.45–3.269)	**3.758 (1.047–13.493)**	4.383 (0.7-27.463)	2.069 (0.204–20.939)
	L	16.9	**1.425 (1.100-1.846)**	1.299 (0.955–1.768)	1.277 (0.835–1.951)	**2.058 (1.067–3.972)**	**2.613 (1.084–6.296)**
		9.9	**1.650 (1.021–2.666)**	1.338 (0.754–2.373)	1.254 (0.573–2.743)	2.230 (0.664–7.496)	2.450 (0.478–12.550)
		6.4	1.004 (0.522–1.932)	0.983 (0.476–2.030)	0.775 (0.314–1.915)	0.732 (0.179–2.993)	0.788 (0.131–4.729)
PTL	H	45.4	1.1060 (0.755–1.621)	0.968 (0.614–1.526)	0.745 (0.405–1.368)	0.772 (0.313–1.907)	0.962 (0.294–3.147)
		43.6	1.154 (0.805–1.653)	1.030 (0.674–1.575)	0.822 (0.471–1.434)	0.879 (0.392–1.969)	1.068 (0.377–3.024)
		42.5	1.179 (0.831–1.673)	1.065 (0.707–1.606)	0.868 (0.511–1.476)	0.942 (0.443–2.006)	1.128 (0.431–2.956)
	L	16.9	0.971 (0.866–1.089)	0.892 (0.780–1.019)	0.855 (0.719–1.017)	0.854 (0.667–1.094)	0.801 (0.585–1.097)
		9.9	0.930 (0.752–1.149)	0.773 (0.605–0.988)	0.713 (0.521–0.974)	0.758 (0.488–1.176)	0.695 (0.398–1.211)
		6.4	0.964 (0.761–1.220)	0.892 (0.691–1.152)	0.974 (0.707–1.340)	1.212 (0.762–1.925)	1.219 (0.676–2.198)
SA	H	45.4	0.903 (0.612–1.333)	0.971 (0.611–1.541)	0.966 (0.522–1.786)	0.852 (0.349–2.080)	0.978 (0.311–3.079)
		43.6	0.867 (0.605–1.244)	0.978 (0.641–1.491)	1.002 (0.576–1.743)	0.855 (0.386–1.893)	0.982 (0.359–2.688)
		42.5	0.849 (0.599–1.204)	0.982 (0.656–1.471)	1.023 (0.605–1.728)	0.860 (0.409–1.808)	0.986 (0.388–2.506)
	L	16.9	1.032 (0.921–1.156)	1.055 (0.923–1.205)	1.021 (0.855–1.218)	0.971 (0.749–1.259)	0.968 (0.695–1.350)
		9.9	1.088 (0.874–1.354)	1.140 (0.881–1.476)	1.095 (0.778–1.541)	1.089 (0.662–1.791)	1.172 (0.619–2.218)
		6.4	1.022 (0.799–1.308)	1.053 (0.798–1.389)	1.007 (0.707–1.435)	1.015 (0.610–1.689)	1.125 (0.586–2.160)
Pre-Eclampsia	H	45.4	0.838 (0.557–1.262)	0.809 (0.496–1.318)	1.033 (0.540–1.977)	1.436 (0.560–3.682)	1.580 (0.471–5.306)
		43.6	0.831 (0.568–1.215)	0.784 (0.501–1.229)	0.979 (0.544–1.764)	1.291 (0.558–2.983)	1.347 (0.465–3.898)
		42.5	0.827 (0.572–1.196)	0.772 (0.502–1.188)	0.951 (0.544–1.663)	1.213 (0.554–2.655)	1.227 (0.46–3.275)
	L	16.9	1.110 (0.985–1.250)	0.997 (0.866–1.146)	0.838 (0.697–1.007)	0.886 (0.682–1.152)	1.018 (0.733–1.413)
		9.9	1.216 (0.964–1.533)	0.990 (0.755–1.297)	0.738 (0.519–1.049)	0.767 (0.466–1.262)	0.807 (0.431–1.511)
		6.4	1.195 (0.923–1.547)	1.034 (0.778–1.374)	0.926 (0.648–1.322)	1.009 (0.605–1.682)	0.906 (0.470–1.746)
Gestational hypertension	H	45.4	1.075 (0.687–1.682)	0.987 (0.580–1.680)	1.151 (0.570–2.321)	1.431 (0.518–3.952)	1.332 (0.363–4.882)
		43.6	1.069 (0.705–1.620)	1.009 (0.619–1.643)	1.220 (0.646–2.304)	1.529 (0.618–3.783)	1.401 (0.446–4.401)
		42.5	1.067 (0.713–1.595)	1.022 (0.64–1.633)	1.261 (0.69–2.305)	1.585 (0.678–3.704)	1.440 (0.499–4.161)
	L	16.9	0.975 (0.856–1.111)	0.952 (0.819–1.107)	**0.791 (0.649–0.965)**	**0.693 (0.519–0.924)**	0.773 (0.537–1.114)
		9.9	1.042 (0.817–1.329)	0.956 (0.720–1.270)	**0.668 (0.458–0.975)**	**0.553 (0.320–0.956)**	0.660 (0.329–1.325)
		6.4	0.950 (0.724–1.246)	0.863 (0.638–1.168)	**0.676 (0.459–0.997)**	0.625 (0.356–1.098)	0.738 (0.358–1.521)

^a^ Physiological Equivalent Temperature (°C)

^b^ High PET values

^c^ Low PET values

**Table 4:** The cumulative relative risks of adverse pregnancy outcomes in intensified thermal stress of PET

Hot Effect (PET)^a^	Lag 0	Lag 0–2	Lag 0–6	Lag 0–13	Lag 0–21
Stillbirth	**1.638 (1.203–2.232)**	1.407 (0.969–2.044)	1.329 (0.817–2.162)	**1.977 (1.004–3.893)**	2.247 (0.941–5.363)
LBW	1.244 (0.763–2.027)	1.144 (0.648–2.022)	1.856 (0.906–3.802)	**2.855 (1.046–7.790)**	2.200 (0.595–8.142)
PTL	0.904 (0.749–1.092)	0.848 (0.675–1.066)	0.759 (0.559–1.030)	0.706 (0.447–1.115)	0.769 (0.420–1.406)
SA	1.099 (0.890–1.358)	0.973 (0.756–1.253)	0.894 (0.645–1.240)	0.950 (0.602–1.501)	0.956 (0.531–1.722)
Pre-Eclampsia	1.017 (0.819–1.263)	1.069 (0.824–1.388)	1.153 (0.823–1.616)	1.396 (0.866–2.252)	1.665 (0.900-3.083)
Gestational hypertension	1.002 (0.787–1.275)	0.925 (0.694–1.234)	0.843 (0.582–1.220)	0.834 (0.497–1.401)	0.874 (0.451–1.693)
**Cold Effect (PET)**^**b**^	**Lag 0**	**Lag 0–2**	**Lag 0–6**	**Lag 0–13**	**Lag 0–20**
Stillbirth	0.931 (0.670–1.292)	0.787 (0.546–1.134)	0.642 (0.407–1.013)	0.633 (0.329–1.217)	0.669 (0.290–1.544)
LBW	0.704 (0.385–1.289)	0.757 (0.394–1.456)	0.607 (0.276–1.333)	0.356 (0.103–1.224)	0.301 (0.067–1.356)
PTL	0.992 (0.823–1.196)	1.001 (0.818–1.224)	1.139 (0.877–1.479)	1.419 (0.947–2.126)	1.521 (0.894–2.589)
SA	0.991 (0.811–1.211)	0.999 (0.800-1.247)	0.987 (0.745–1.308)	1.045 (0.695–1.571)	1.162 (0.687–1.964)
Pre-Eclampsia	1.077 (0.874–1.327)	1.038 (0.828–1.301)	1.105 (0.833–1.465)	1.138 (0.746–1.736)	0.890 (0.508–1.559)
Gestational hypertension	0.974 (0.782–1.213)	0.906 (0.710–1.157)	0.855 (0.626–1.167)	0.903 (0.571–1.428)	0.954 (0.523–1.739)

^a^ The cumulative effects of hot thermal stress on adverse pregnancy outcomes, with 99th percentile of PET (45.4 °C) relative to 75th percentile of PET (39.1 °C)

^b^ The cumulative effects of cold thermal stress on adverse pregnancy outcomes, with 1st percentile of PET (6.4 °C) relative to 25th percentile of PET (16.9 °C)

## Discussion

Pregnant mothers and growing embryos are among the populations likely to be susceptible to climate change. Climate change may have a significant impact on the health and survival of future human generations [37].

The purpose of the present study was to determine the relation between PET index with adverse pregnancy outcomes including stillbirth, low birth weight, preterm labor, spontaneous abortion, preeclampsia and gestational hypertension in Ahvaz city.

In the present study, the results showed that high values of PET (the 99th percentile) and hot thermal stress according to the PET index, increased the risk of stillbirth. Similarly, Asamoah et al., studied the association between ambient heat exposure and stillbirths in Ghana; and showed that for every one degree increase in wet-bulb globe temperature (WBGT), the risk of stillbirth or miscarriage increased 12–15 %. Although this increase was not statistically significant, but the small range of WBGT changes over the months and years may have hidden the real impact of high temperature levels [38]. Other studies have confirmed the effect of heat stress on stillbirth as well. Rammah et al., (2019), conducted a study to examine associations between increases of apparent temperature and stillbirths during the warm season (May–September), among 708 women, from 6 days before stillbirth, in Harris County, Texas, from 2008 to 2013. The results showed that a 10 °F increase in apparent temperature in the week before delivery was related to a 45 % increased risk of stillbirth (adjusted OR = 1.45, 95 % confidence interval (CI): 1.18, 1.77) [7]. Likewise, another study in California showed for every 10 °F increase in apparent temperature during the warm season, the risk of stillbirth increased by 10.4 % after 2–6 days lag, while there was no significant relation between temperature and stillbirth during the cold season [10]. A study conducted by Strand et al., (2011), in Brisbane city, Australia, during 2005–2009, reported that high ambient temperature during the last 4 weeks of pregnancy increased the risk of stillbirth compared to mothers exposed in only the last week. These results suggested that exposure to hot thermal stress should be considered not only in the last week of pregnancy, but also in longer periods. Strand et al. also found that increased temperatures increased the probability of stillbirth before 36 weeks’ gestation, and the risk of stillbirth associated with high temperatures was greater in early gestation [39]. Studies have reported the effects of cold weather as well. Ha et al., (2017) conducted a study on 22,3375 single births in 12 US cities between 2002 and 2008, and reported that chronic exposure to local hot (OR: 3.71, 95 % CI: 3.07–4.47) and cold (OR: 4.75, 95 % CI: 3.95–5.71) weather relative to mild weather, throughout pregnancy was associated with an increased risk of stillbirth. Also, for every 1 °C increase in temperature in the week before delivery, in hot seasons (May-September), the risk of stillbirth increased by 6 % (95 % CI: 3–9 %); and it seemed that only warm weather had a significant acute association with the risk of stillbirth, especially in the week before delivery [40]. Bruckner et al. (2014) conducted a retrospective cohort study in Uppsala, Sweden, from 1915 to 1929, and showed that cold weather increased the risk of stillbirth, but contrary to our findings warm weather had a protective effect, and for every 1 °C increase in temperature throughout pregnancy, the risk of stillbirth decreased by 8 % [41].

The mechanism of effect of temperature on stillbirth is unclear, but it seems that changes in hormonal levels and increase in fetal circulation demand in the mother may increase her susceptibility to heat disorders. High thermal stress may cause water shortages in the mother and be harmful to the fetus. Inadequate fluids in the mother can reduce blood volumes and cause uterine contractions and stillbirth [42]. Low asymptomatic levels of thermal stress in the mother might theoretically result in increased shunting of blood to the periphery, as a heat dissipation mechanism. This can result in altered placental and umbilical blood perfusion and reduced heat exchange with the fetus [43]. Also, extreme temperatures can lead to compromised endothelial function as well as rheological changes, both of which may affect blood pressure and blood viscosity [44, 45]. These responses may ultimately alter maternal–fetal exchange and disturb fetal growth and survival [46].

In relation to LBW, both high values of PET in the 90th, 95th and 99th percentiles and low values of PET in the 5th and 25th percentiles, increased the risk of LBW throughout all lags. Also, hot thermal stress of PET increased the risk of LBW. Similarly, other studies confirm the effect of hot and cold weather on LBW. A retrospective observational study, conducted by Sun et al., (2019), based on data from nearly 30 million births in 403 cities in the US, found that high temperature (> 90th percentile) throughout pregnancy was associated with increased risk of small for gestational age (SGA) [OR: 1.041 (95 % CI: 1.029–1.054)] and reduced birth weight [− 15 g (95 % CI: −17g_−13 g)]. On the other hand, low temperature (≤ 10th percentile) was not associated with SGA [OR = 1.003 (95 % CI: 0.991, 1.015)] risk, but a slight decrease in birth weight [− 6 g (95 % CI: −8g_−4 g)] was observed [47].

Grace et al., (2015), conducted a study in 19 African countries, on nearly 70,000 births, over 20 years, and noticed that with increased warm days and reduced rainfall at any point in pregnancy, a significant increase in LBW was observed in sub-Saharan Africa [13]. Also, Kloog et al., (2015) conducted a study in Massachusetts, and found that temperature had an inverse relation with birth weight, and for each IQR increase in temperature (8.4 °C) during the last 3 months of pregnancy, mean birth weight decreased by 16.7 g [48].

On the other hand, Hartig and Catalano (2013) stated that the risk of very low birth weight (< 1500 g) was related to ambient cold temperatures, during the cold summers of Sweden [49]. Contrary to our results, Bruckner et al., (2014), in a study in Uppsala, Sweden found no relation between ambient temperature and birth weight [41]. Researchers think high and low thermal stress are associated with oxidative stress and systemic inflammation [50, 51]; and high thermal stress leads to the release of the heat shock proteins (HSPs) including HSP-70 in humans [52]. Increased HSP-70 levels are effective in causing intrauterine growth restriction (IUGR) and may lead to a range of adverse pregnancy outcomes [53, 54]. In addition, higher or lower than average temperatures are also directly associated with changes in blood viscosity and uterine blood flow [55, 56] which can affect the growth of the fetus.

Concerning preterm labor, the results of our study did not show any significant relation between the effect of PET and PTL. Similarly, two cohort studies suggested that there was no increase or decrease in the risk of preterm birth among those who were exposed to short-term cold temperatures throughout their pregnancy [57, 58].

However, some studies have stated that both hot and cold weather, affect PTL. For example, in Sabzevar, Iran, an increased risk of preterm birth at both very hot and very cold temperatures especially at 0 till 8 lag days was observed [59]. Also, Li et al., (2018), reported that high and low temperature in the second and third trimesters of pregnancy increased the risk of stillbirth and preterm labor, respectively, in Brisbane, during 1993–2013. The results also showed that the effects of low temperature, were stronger, and the effects of high temperature, were weaker for both preterm delivery and stillbirth over time [60].

In relation to gestational hypertension, the results of this study showed that low levels of PET in the 1st, 5th and 25th percentiles reduce the risk of hypertension. In contrast, the study done by Melo et al., (2014), on 26,125 admitted pregnant women, between 2000 and 2006 in Recife, Brazil, reported that during the cold months, the incidence of hypertension disorders in pregnancy significantly increased, and the lowest mean monthly incidence was in February (9.95 %) and the highest in August (21.54 %) [19]. Some longitudinal studies have examined seasonal variations in blood pressure during pregnancy, and found that blood pressure in winter and summer months are at their highest and lowest, respectively [17, 18]. But Wellington and Mulla (2012), in Texas, observed a low prevalence of hypertension in pregnant women in winter compared to autumn [61]. Based on the studies mentioned above and the results of a systematic study by TePoel et al. (2011), hypertension in pregnancy occurs mostly in winter [62]. There are various possible mechanisms involved in the effect of cold weather on hypertension, including increased risk of seasonal infections [63], physiological responses to cold, decreased physical activity [64, 65], and the reduction of vitamin D levels in plasma [17, 66]. Sunlight is the main source of vitamin D [67]. Researchers think the reduction of this vitamin is associated with an increased risk of hypertension [68]. Since Ahvaz has hot summers and cool winters, people prefer to do more physical activity and are more exposed to outside air and sunlight in the winter. Therefore, possibly due to more physical activity and normal levels of vitamin D, cold weather has a protective effect on hypertension in pregnancy in this city.

In this study, no significant relation was found between temperature changes and preeclampsia. Similar to this a 36-month research on 11,958 newborns in Mississippi, found no association between the incidence of hypertension in pregnancy and seasonal variation [69]. However, a study from the northeast of Iran found a significant association between monthly temperature changes and preeclampsia prevalence; and the highest prevalence of preeclampsia was seen in summer, especially in September (11.1 %) and August (10.3 %) and the lowest prevalence was seen in winter and early spring, especially in January (5.6 %) and April (5.7 %) [70]. Tam et al., conducted a study in Hong Kong, between 1995 and 2002, and found that women who were pregnant in the summer and had been exposed to higher temperatures were more likely to have preeclampsia [14]. On the other hand, an analysis about the monthly changes of preeclampsia by Shental et al., in Negev, Israel showed that the lowest and highest incidence of preeclampsia occurred in the hottest and coldest months, respectively [71]. However, these findings are inconsistent and more studies with more accurate exposure methods are required.

Although, the relation between temperature and some adverse pregnancy outcomes has been studied before in Iran, the present study included abortion and gestational hypertension for the first time. This study comprehensively examined the effect of PET index on six adverse pregnancy outcomes.

This study had some limitations, including the lack of some health and individual data such as maternal weight, body mass index, and physical activity. Also, this was an ecological study, which means that these results are at population level and cannot be generalized to the individual level. Air pollution parameters had been measured at population level and we were not aware of the individual exposure status.

## Conclusions

The results of this study showed that hot and cold thermal stress may be associated with increased risk of stillbirth, and LBW. Increasing our understanding about the link between temperature changes and adverse pregnancy outcomes in societies sensitive to climate change is essential for improving maternal and fetal health. Pregnant women should be aware of the risk of high and low temperatures for unborn babies and should be encouraged to protect themselves from excessive thermal stress.

## Data Availability

Data are available from the corresponding author upon reasonable request..

## Abbreviations

PET: Physiological equivalent temperature
LBW: Low birth weight
PTL: Preterm labor
SA: Spontaneous abortion
DLNM: Lag non-linear models
NO_2_: Nitrogen dioxide
SO_2_: Sulfur dioxide
PM_10_: Particles with a diameter of less than 10 micrometers
EM: Expectation maximization
AIC: Akaike information criteria
SGA: Small for gestational age
IUGR: Intrauterine growth restriction
WBGT: Wet-bulb globe temperature
MEMI: Munich energy balance for individuals
